# Anti–nerve growth factor therapy attenuates cutaneous hypersensitivity and musculoskeletal discomfort in mice with osteoporosis

**DOI:** 10.1097/PR9.0000000000000652

**Published:** 2018-04-10

**Authors:** Miyako Suzuki, Magali Millecamps, Seiji Ohtori, Chisato Mori, Laura S. Stone

**Affiliations:** a Center for Preventive Medical Sciences, Chiba University, Chiba, Japan; b The Alan Edwards Centre for Research on Pain, McGill University, Montreal, QC, Canada; c Faculty of Dentistry, McGill University, Montreal, QC, Canada; Departments of d Orthopedic Surgery and; e Bioenvironmental Medicine, Graduate School of Medicine, Chiba University, Chiba, Japan; f Department of Pharmacology and Therapeutics, Faculty of Medicine, McGill University, Montreal, QC, Canada

**Keywords:** Osteoporosis, Osteoporosis-related pain, Anti-NGF therapy, OVX-induced mouse model

## Abstract

**Introduction:**

The prevalence of osteoporosis is increasing with the aging population and is associated with increased risk of fracture and chronic pain. Osteoporosis is currently treated with bisphosphonate therapy to attenuate bone loss. We previously reported that improvement in bone mineral density is not sufficient to reduce osteoporosis-related pain in an ovariectomy (OVX)-induced mouse model of osteoporosis, highlighting the need for new treatments. Targeting of nerve growth factor (NGF) with sequestering antibodies is a promising new direction for the treatment of musculoskeletal pain including back pain and arthritis. Its efficacy is currently unknown for osteoporotic pain.

**Objective:**

To investigate the efficacy of anti-NGF antibody therapy on osteoporotic pain in an OVX-induced mouse model.

**Methods:**

Ovariectomy- and sham-operated mice were injected with an anti-NGF antibody (10 mg/kg, intraperitoneally, administered 2×, 14 days apart), and the effect on behavioural indices of osteoporosis-related pain and on sensory neuron plasticity was evaluated.

**Results:**

Treatment with anti-NGF antibodies attenuated OVX-induced hypersensitivity to mechanical, cold, and heat stimuli on the plantar surface of the hind paw. The OVX-induced impairment in grip force strength, used here as a measure of axial discomfort, was partially reversed by anti-NGF therapy. No changes were observed in the rotarod or open-field tests for overall motor function and activity. Finally, anti-NGF treatment attenuated the increase in calcitonin gene-related peptide–immunoreactive dorsal root ganglia neurons observed in OVX mice.

**Conclusion:**

Taken together, these data suggest that anti-NGF antibodies may be useful in the treatment of prefracture hypersensitivity that is reported in 10% of patients with osteoporosis.

## 1. Introduction

Osteoporosis-related pain can result from local fractures,^40^ but is also seen in the absence of observable bone trauma.^30^ We recently reported cutaneous hypersensitivity and deep musculoskeletal pain in a mouse model of ovariectomy (OVX)-induced osteoporosis.^37^ Studies investigating bone pain have demonstrated that increased osteoclast activity produces acidic microenvironments within the bone and activation of primary afferent terminals.^24,25^ This activity promotes the release of proinflammatory mediators including nerve growth factor (NGF), which increases expression of the neuropeptide calcitonin gene-related peptide (CGRP) in dorsal root ganglia (DRG), and contributes to sensory hypersensitivity.^33,38,43^

In clinical studies, anti-NGF therapy has efficacy against skeletal pain arising from osteoarthritis and low back pain.^1,18,29,39^ Anti-NGF therapy is untested in osteoporosis-related pain. The aim of this study was to examine the efficacy of anti-NGF antibody therapy against cutaneous hypersensitivity, deep musculoskeletal pain, and physical function in a mouse model of OVX-induced osteoporosis.

## 2. Materials and methods

### 2.1. Animals

Female C57BL6 mice (Charles River Laboratories, Montreal, QC, Canada) were housed in an environmentally controlled facility with free access to food and water. Mice were randomly assigned to the sham-operated (n = 10) or OVX (n = 20) groups. All experiments were approved by the Animal Care Committee at McGill University and conformed to the ethical guidelines of the Canadian Council on Animal Care and the International Association for the Study of Pain (IASP).^45^

### 2.2. Surgical procedures and bone mineral density

Surgical procedures were performed at 5 to 7 weeks of age as described.^17,28,37^ In the sham-operated group, ovaries were exposed using the identical procedure but were left intact. Bone mineral density (BMD) was measured by dual-energy x-ray absorptiometry densitometry.

### 2.3. Behavioral procedures

Animals were habituated in the apparatus for 60 minutes before testing. Testing was between 9:00 am and 3:00 pm.^21,37^ The experimenter was blind to surgical and treatment groups, all testing was performed in parallel, and all animals were included. Cutaneous sensitivity, musculoskeletal discomfort, and physical function were each evaluated on different test days.

#### 2.3.1. Cutaneous sensitivity to mechanical, cold, and heat stimuli

Mechanical sensitivity was assessed using the von Frey up–down method.^3^ Cold sensitivity was evaluated by (1) nociceptive behaviors following a drop of acetone to the left hind paw^4,21,22^ and (2) the latency to the first brisk hind paw withdrawal from a 4°C plate (cutoff 30 seconds; Hot/Cold Plate, 35100, Ugo Basile, Varese, Italy).^4^ Heat sensitivity was detected as the latency to withdrawal from a radiant heat stimulus (cutoff 22.7 seconds; IITC Life Science Inc, Woodland Hills, CA).^10^

#### 2.3.2. Deep musculoskeletal discomfort and physical function

The grip test assay measuring resistance to anteroposterior stretching as a behavioral index of deep musculoskeletal pain (Stoelting Co, Wood Dale, IL).^15,41^ Physical function was evaluated using the rotarod (Ugo Basil SRL, Varese, Italy) and a 5-minute open-field test.^13,21^

### 2.4. Anti–nerve growth factor treatment

Animals received either anti-NGF or vehicle (OVX/anti-NGF, OVX/Vehicle, and Sham/Vehicle; n = 10 per group). Two 10 mg/kg doses of anti-NGF mouse monoclonal antibody (Exalpha Biologicals Inc, Shirley, MA) were administered through intraperitoneal (i.p.) injection on days 0 and 13. Vehicle treatment (0.01 mL/g i.p. sterile saline) followed the same schedule.

### 2.5. Immunohistochemistry

Immunohistochemistry was performed as previously described.^23^ Ten micrometer cryostat sections of upper (L1-3) and lower (L4-6) DRG were incubated with anti-CGRP (1:500, Cat#BML-CA1137, Lot#01101327; Enzo Life Sciences, Farmingdale, NY) and Guinea pig-derived anti-NPY (1:500; Cat#AB10341, Lot#GR12360; Abcam, Tokyo, Japan), and visualized with Cy3-goat anti-sheep IgG and Cy2-goat anti-guinea pig IgG (1:500; Jackson Immuno Research, West Grove, PA) with an Olympus BX-5. Ten sections each from the upper and lower DRG per mouse were randomly selected for analysis. The proportion of CGRP-immunoreactive (ir) and NPY-ir neurons among total DRG neurons was determined.

### 2.6. Data analyses

Body weight and BMD were analyzed by a 1-tailed unpaired *t* test or 1-way analysis of variance (ANOVA) followed by the Tukey test. Behavioural measures between baseline and day 28 were analyzed by 2-way ANOVA with group and time as factors, followed by the Dunnet multiple comparisons test. Immunohistochemistry and the day 56 behaviour (only a subset was retested on day 56) were analyzed by 1-way ANOVA followed by the Tukey test. The statistical analyses were performed using GraphPad Prism 6 software.

## 3. Results

### 3.1. Effects of ovariectomy and anti–nerve growth factor treatment on body weight and bone mineral density

**Figure 1.**
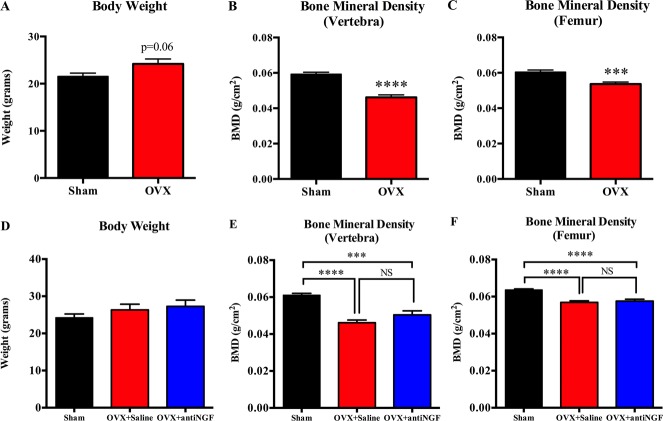
Effects of ovariectomy and anti-NGF treatment on body weight and bone mineral density. Ovariectomized (OVX) mice tended towards an increase in body weight compared with sham-operated mice (A). Vertebral (B) and femoral (C) bone mineral density are significantly decreased in OVX-operated compared with sham-operated mice 8 weeks after surgery. Anti-NGF treatment had no effect on body weight (D), vertebral (E), or femoral (F) bone mineral density. Sham + saline mice (black bars, left columns), OVX + saline mice (red bars, middle columns), and OVX + anti-NGF (blue bars, right columns). Data are expressed as mean ± SEM. ****P < 0.0001, ***P < 0.001; (A–C) OVX-operated vs sham-operated, 1-tailed unpaired *t* test (equal variances). (D–F) OVX + saline or OVX + anti-NGF vs sham + saline; 1-way ANOVA, followed by the Tukey multiple comparisons. ANOVA, analysis of variance; BMD, bone mineral density; NGF, nerve growth factor. NS, not significant.

Ovariectomy mice trended towards an increase in body weight compared with sham-operated mice (Fig. 1A, *P* = 0.06). Vertebral and femoral BMD were significantly lower in OVX compared with sham-operated mice (Fig. 1B, C). Anti-NGF treatment in OVX mice had no effect (Fig. 1D–F).

### 3.2. Efficacy of anti–nerve growth factor treatment on behavioral indices of osteoporosis-related cutaneous hypersensitivity, deep musculoskeletal pain, and physical function

**Figure 2.**
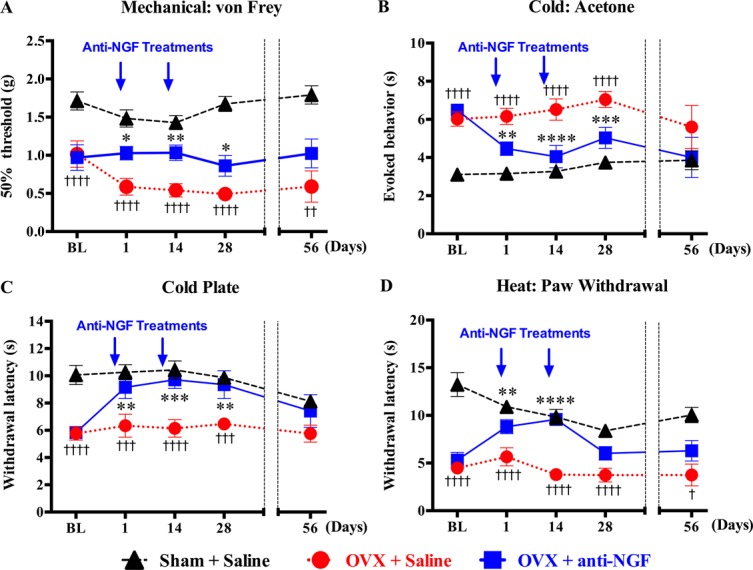
Efficacy of anti-NGF treatment on behavioral indices of osteoporosis-related cutaneous hypersensitivity. Hypersensitivity to cutaneous plantar mechanical stimuli (von Frey assay) (A), cold (acetone test) (B) and cold plate tests (C), and heat (radiant heat hind paw withdrawal assay) (D) develop by 8 weeks after ovariectomy (baseline, BL). Anti-NGF (10 mg/kg) was injected two times 1 day before behavioral testing on day 1 and day 14. X-axis is the elapsed days from the first injection. Anti-NGF treatment significantly reversed cutaneous mechanical (A), cold (B and C), and heat hypersensitivity (D) in OVX mice. Nerve growth factor efficacy was gone by day 56. Arrow = anti-NGF injection; BL, baseline, 8 weeks after ovariectomy or sham surgeries; OVX; ovariectomized. Data are expressed as mean ± SEM. †*P* < 0.05; ††*P* < 0.01; †††*P* < 0.001; ††††*P* < 0.0001; OVX + saline vs sham + saline; *P < 0.05; **P < 0.01; ***P < 0.001; ****P < 0.0001; OVX + saline vs OVX + anti-NGF. BL to 28 days; 2-way ANOVA, repeated-measures ANOVA (factors = group × time), followed by the Dunnet multiple comparisons test, at 56 days; 1-way ANOVA followed by the Dunnet multiple comparisons test. ANOVA, analysis of variance; NGF, nerve growth factor.

Hypersensitivity to mechanical, cold, and heat (Fig. 2A–D, baseline) was fully developed 8 weeks after OVX. Anti-NGF treatment (injected days 0 and 13) reversed mechanical (Fig. 2A) and cold (Fig. 2B, C) throughout the 28-day study. Hypersensitivity to heat was reduced at 1 and 14 days but not at 28-day post-anti-NGF (Fig. 2D).

**Figure 3.**
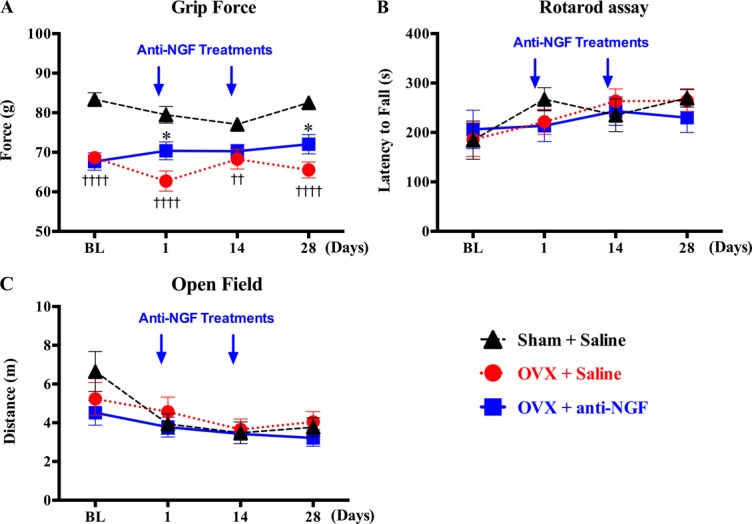
Efficacy of anti-NGF treatment on behavioral indices of osteoporosis-related deep musculoskeletal pain and physical function. In the grip force strength assay (A), the mice were held by the tail while gripping a bar and were gently stretched until the point of release, the force at release in grams is used here as a behavioral index of deep musculoskeletal pain. The reduced strength was observed at baseline, 8 weeks after surgery in OVX mice (A). Anti-NGF treatment had an effect at several time points on OVX-operated mice compared with saline (A). In rotarod assay, locomotor capacity was measured with an accelerating rotarod. The experimental end point occurs when the animal falls off the cylinder. In an open-field test, mice were individually placed into the center of a transparent open-field apparatus (24 × 24 cm) and their spontaneous behavior was videotaped for 5 minutes to assess general motor activity. The total distance covered during the 5-minute test period was analyzed using ANY-maze software (Stoelting) by an observer blinded to the experimental conditions. No changes were observed in the rotarod (B) or open-field tests (C) for physical function after ovariectomy or anti-NGF treatment. Arrow = anti-NGF injection, BL; baseline, at 8 weeks after ovariectomy or sham surgeries, OVX; ovariectomized. Data are expressed as mean ± SEM. ††*P* < 0.01; ††††*P* < 0.0001; OVX + saline vs sham + saline, *P < 0.05; OVX + saline vs OVX + anti-NGF, 2-way ANOVA, repeated-measures ANOVA (factors = group × time), followed by the Dunnet multiple comparisons test. ANOVA, analysis of variance; NGF, nerve growth factor.

Grip strength was decreased in OVX 8 weeks after surgery (Fig. 3A, baseline). Anti-NGF treatment had significant effects at 1 day and 28 days in OVX-operated mice (Fig. 3A). No effects of OVX surgery or anti-NGF treatment were observed in the rotarod and open-field assays (Fig. 3B, C).

### 3.3. Effect of anti–nerve growth factor treatment on expression of calcitonin gene-related peptide-ir and NPY-ir neurons in dorsal root ganglia

**Figure 4.**
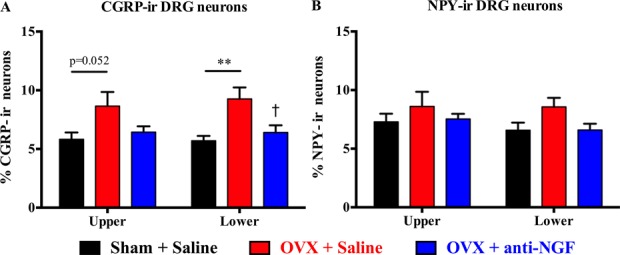
Effect of anti-NGF treatment on expression of CGRP-ir and NPY-ir neurons in dorsal root ganglia. The percentage of CGRP-ir (A) or NPY-ir (B) neurons in the upper lumbar (L1–L3) and lower lumbar (L4–L6) DRG was evaluated in sham + saline mice (black bars, left columns, OVX + saline mice red bars, middle columns) and OVX + anti-NGF (blue bars, right columns). Calcitonin gene-related peptide–ir neurons in the lower lumbar DRG in OVX + saline with significantly increased compared with sham + saline mice, and anti-NGF treatment significantly decreased the expression of CGRP-ir neurons in OVX mice (A). In the upper lumbar DRG, CGRP-ir neurons just tended to be higher than sham-operated mice, but there was no significant difference between the OVX group with vehicle and with anti-NGF (A). In the expression of NPY-ir neurons in both upper and lower lumbar DRG, no effects of OVX-surgery or anti-NGF treatment were observed (B). Data are expressed as mean ± SEM. **P < 0.01; OVX + saline vs sham + saline; †*P* < 0.05; OVX + saline vs OVX + anti-NGF, 1-way ANOVA, followed by the Tukey multiple comparisons. ANOVA, analysis of variance; CGRP, calcitonin gene-related peptide; DRG, dorsal root ganglia; NGF, nerve growth factor; NPY, neuropeptide-Y; OVX, ovariectomized.

The significant increase in CGRP-ir in DRG neurons in OVX mice was attenuated by anti-NGF treatment (Fig. 4A). NPY-ir in DRG neurons was not affected by OVX or anti-NGF (Fig. 4B).

## 4. Discussion

Here, we demonstrate attenuation of OVX-induced cutaneous hind paw hypersensitivity, deep musculoskeletal pain, and increased CGRP-ir after anti-NGF treatment. This is the first report to demonstrate efficacy of anti-NGF therapy in an osteoporosis model.

### 4.1. Effects of anti–nerve growth factor therapy on behavioral indices of osteoporotic pain

In osteoporotic mice, 2 administrations of anti-NGF therapy (days 0 and 13) reduced mechanical and cold hypersensitivity for up to 28 days, suggesting that the effects are long lasting. Heat hypersensitivity was reversed on days 1 and 14 but not day 28, suggesting a more acute effect. Anti-NGF partially reversed impaired grip strength, an index of deep musculoskeletal discomfort. Neither OVX nor treatment affected the rotarod and open-field assays for physical function; consistent with the emergence of physical disability after fracture or physical deformity rather than osteoporosis per se. The efficacy of anti-NGF therapy in osteoporosis-related pain is consistent with efficacy in other skeletal pain-related animal models including malignant bone cancer,^8^ knee arthritis,^5^ and bone fracture pain.^6,12,16,35^ Together, these studies suggest a critical role for NGF in driving bone- and joint-related pain.

### 4.2. Potential mechanisms: effects of anti–nerve growth factor therapy on sensory neurons and bone homeostasis

We previously reported increased innervation of bone marrow and neuroplasticity in the spinal cord in rodent models of osteoporosis-induced pain, suggesting that osteoporosis-related pain is associated with nerve sprouting and sensitization.^14,33,38,44^ Here, the proportion of CGRP-ir DRG neurons was increased after OVX. The acidic microenvironment^24,25,31–33,38^ and secretion of local proinflammatory cytokines^34^ may contribute to increased CGRP expression, resulting in neurogenic inflammation and hypersensitivity.^24,25,31–33,38^

In addition to roles in development and maintenance of sensory and sympathetic neurons,^19^ NGF plays a role in bone differentiation^27^ and bone fracture repair.^11^ Most sensory nerve fibers in bone express the high-affinity NGF receptor TrkA.^8,12,26,36^ In osteoporosis, proinflammatory cytokines, such as TNFα and IL-1β, increase in bone marrow; NGF regulates these substances.^20^ The efficacy of anti-NGF therapy in musculoskeletal-related pain may be mediated, in part, by a reduction in NGF-maintained inflammation.

### 4.3. Potential limitations and future directions

First, we investigated the effects of 1 concentration of anti-NGF antibodies, administered through 1 route (i.p.) with 1 dosing schedule following a protocol efficacious in mice with fracture or neuropathic pain.^16,42^ Additional studies are needed to optimize treatment parameters, and conclusions regarding potency, efficacy, and duration of action are premature. Second, because the literature is unclear regarding specific characteristics of osteoporosis-related pain in humans, the behavioural end points used here should be considered as proxy measures. Third, the study was performed in females; extension to male subjects is an important next step. Fourth, anti-NGF was only tested in OVX mice. However, no effects of this treatment have been reported in other preclinical studies on control animals^9^ and in clinical studies on healthy volunteers^7^; phase III clinical studies are ongoing.^2^

Finally, here, we observed changes in neuropeptide expression in DRG; future studies should directly examine sensory innervation in osteoporotic bone and osteoporosis-related changes in skin to fully understand sensory-bone interactions.

### 4.4. Conclusions

Here, we demonstrate anti-NGF efficacy in the OVX mouse model of osteoporosis-related pain. These data implicate NGF as a driver of long-term osteoporotic pain and suggest that anti-NGF treatment may be a useful therapy for this population.

## Disclosures

The authors have no conflict of interest to declare.

This work was supported by the Canadian Institutes of Health Research Grants MOP102586, MOP126046, and MOP142291 to M. Millecamps and L.S. Stone.
